# Invasive Group A Streptococcal Infection in High School Football Players, New York City, 2003

**DOI:** 10.3201/eid1101.040559

**Published:** 2005-01

**Authors:** Susan E. Manning, Elsie Lee, Maribeth Bambino, Joel Ackelsberg, Don Weiss, Chiminyan Sathyakumar, John Kornblum, Oxiris Barbot, Dwight Johnson, Edward L. Kaplan, Marcelle Layton

**Affiliations:** * Centers for Disease Control and Prevention, Atlanta, Georgia, USA; † New York City Department of Health and Mental Hygiene, New York, New York, USA; ‡ Montefiore Medical Center, Department of Infectious Diseases, Bronx, New York, USA; § World Health Organization Collaborating Center for Reference and Research on Streptococci, Minneapolis, Minnesota, USA

**Keywords:** Group A Streptococcus, GAS, invasive GAS, competitive sports, antibiotic prophylaxis for GAS, athletic teams, dispatch

## Abstract

After being notified that 2 high school football teammates were hospitalized with confirmed or suspected invasive group A streptococcal infections, we conducted an investigation of possible spread among other team members. This investigation highlights a need for guidelines on management of streptococcal and other infectious disease outbreaks in team sport settings.

Group A *Streptococcus* (*S. pyogenes*; GAS), a bacterium commonly found on the oropharynx and skin, causes infections ranging from relatively mild and localized to invasive and potentially life-threatening (*1*). In October 2003, the New York City Department of Health and Mental Hygiene was notified of 2 high school varsity football teammates hospitalized on the same day, one with culture-confirmed GAS septic thrombophlebitis and another with suspected GAS pyoderma.

## Case Studies

A 17-year-old male high school student (patient 1) visited the emergency department of a local hospital in Bronx, New York, on October 20; he had bilateral groin pain that had begun 2 days previously, shortly after he played in a high school football game. He complained of pain with swallowing but denied fever or chills. His oropharynx was erythematous but without tonsillar exudate. Abdominal examination showed suprapubic tenderness and voluntary guarding. His thighs were tender bilaterally. A rapid streptococcal antigen throat swab test result was positive. Computed tomographic (CT) scan of the abdomen suggested acute appendicitis, and CT and duplex ultrasound confirmed a right external iliac vein thrombus. Exploratory laparotomy showed a normal appendix and no evidence of acute pathology. Blood cultures were positive for GAS. He was treated with antimicrobial agents and anticoagulation for septic external iliac thrombus.

Another 17-year-old male high school student (patient 2) from the same football team was hospitalized at the same hospital on October 20 for a fluctuant right leg mass. Two weeks previously, he had sought medical care for sore throat and erythematous skin overlying his right posterior calf, where he was hit by a helmet during a game. A rapid streptococcal antigen throat swab test result was positive, and he was treated with amoxicillin for 10 days. Two days before admission, he noticed increased swelling and a blister on his right calf. He was afebrile, and an oropharyngeal examination was normal. His right lower leg was swollen posteriorly from behind the knee to the ankle. Blood culture was negative. The right calf swelling was drained, and ≈1 L of purulent fluid was removed. Results of a rapid streptococcal antigen test of the aspirated fluid were positive, but Gram stain and cultures were negative.

On October 24, 2003, hours before the team’s homecoming game, the New York City Department of Health and Mental Hygiene was notified about these hospitalized teammates. The health department also learned that other varsity teammates recently had sore throats and skin lesions. Given the close physical contact and sharing of equipment and water bottles among players, the health department launched an epidemiologic investigation that evening at the high school. Teammates reporting symptoms consistent with GAS infection were to be excluded from play. However, since none of the players reported having symptoms consistent with acute GAS disease, the homecoming game was allowed to proceed.

A screening questionnaire was designed to identify persons with current or recent throat and skin infections and risk exposures for GAS. Thirty-three varsity players and 5 coaches were interviewed. Throat cultures from the 33 varsity players and 5 coaches were screened for GAS by using standard methods (*2*). Isolates from positive culture specimens were characterized by using pulsed-field gel electrophoresis (PFGE) with *Sma* I and *Sfi* I enzymes (*3*). GAS isolates were tested for susceptibility to chloramphenicol, clindamycin, erythromycin, penicillin G, tetracycline, and vancomycin by using standard agar disc diffusion (Kirby-Bauer, Remel, Lenexa, KS).

Positive isolates were characterized at the World Health Organization Collaborating Center for Reference and Research on Streptococci at the University of Minnesota Medical School. Isolates were serologically grouped and further subtyped by opacity factor, T-agglutination pattern, and *emm* sequencing (*2*,*4*). To prevent additional cases of GAS, antimicrobial prophylaxis with either penicillin and rifampin or azithromycin was recommended for all varsity teammates and coaches.

The 33 varsity players and 5 coaches ranged in age from 15 to 55 years (median age 17 years). None reported symptoms consistent with acute GAS infection. Among the 38 throat cultures obtained, 3 (8%) were positive for β-hemolytic GAS (Table). The only reported exposure to ill persons was contact with the 2 patients.

**Table:** Characterization of group A *Streptococcus* isolates from high-school varsity and junior varsity football players, New York City, 2003

Specimen origin	Site	Antimicrobial susceptibility*	PFGE†	M/OF†/*emm* type
Varsity player				
A‡	Blood	Susceptible to all antimicrobial agents tested		82
B	Throat	Resistant to erythromycin, susceptible to all others	Unrelated§	75
C	Throat	Susceptible to all antimicrobial agents tested	Unrelated	6
D	Throat	Intermediate to tetracycline, susceptible to all others	Indistinguishable	82
Junior varsity player				
E	Throat	Susceptible to all antimicrobial agents tested	Unrelated	89
F	Throat	Susceptible to all antimicrobial agents tested	Unrelated	44/61
G	Throat	Susceptible to all antimicrobial agents tested	Unrelated	28
H	Throat	Susceptible to all antimicrobial agents tested	Unrelated	118

*Antimicrobial agents tested: chloramphenicol, clindamycin, erythromycin, penicillin G, tetracycline, vancomycin. †Entries represent putative genetic relatedness to the case-patient no. 1 strain A based on *Sma*I and *Sfi*I DNA restriction patterns by using categories as defined by Tenover et al. (*5*). The results obtained with *Sfi* I correlated completely with the results obtained with *Sma*I; PFGE, pulse-field gel electrophoresis; OF, opacity-factor. ‡Case-patient #1. §Specimen B was nontypable with *Sma*I but was typable with *Sfi*I.

One GAS isolate from an asymptomatic player (specimen C) was *emm*-type 6, and another (specimen B) was *emm*-type 75. The isolate from a third asymptomatic player (specimen D) was indistinguishable from that of the blood culture (specimen A) from patient 1 by PFGE analysis and *emm* sequencing (type 82).

During the investigation on October 24, the New York City Department of Health and Mental Hygiene learned that the junior varsity team shared equipment and water bottles with varsity players but did not have the same close, skin-to-skin contact with the patients as did varsity teammates. Therefore, the health department screened all junior varsity players on October 27 but provided treatment only for those whose throat cultures were GAS-positive. Among 51 junior varsity team members and 3 coaches screened, 4 (7%) had GAS-positive throat cultures (Table). Two of the 4 reported current or recent symptoms, including headache and stomachache. The other 2 denied having symptoms or contact with ill persons. The positive isolates from junior varsity players were unrelated by PFGE or *emm* typing to strains from other varsity or junior varsity players, including patient 1.

## Conclusions

GAS infections are typically spread through contact with mucus from infected persons or with infected skin lesions (*6*). GAS infections in athletic settings could be transmitted by person-to-person contact, airborne or droplet spread, or exposure to shared-use items (*7*).

In 2002, guidelines for preventing invasive GAS among household contacts of case-patients were published (*8*). These guidelines address the management of household contacts but not of other types of close, physical contacts (e.g., athletic teams). General guidelines exist regarding preventing infectious diseases in athletic settings (*7*,*9*). However, no specific recommendations have been published regarding prevention strategies after invasive GAS cases have been identified among athletes in contact sports such as football, a setting where spread of GAS is possible because of repetitive and forceful skin-to-skin contact and resultant trauma.

Although the recommendations advise against routine screening and prophylaxis for household contacts, prophylaxis is recommended in certain situations where host factors are associated with increased risk for invasive disease (*8*). We hypothesized that teammates are at least as likely to share drinks from common sources and have close, skin-to-skin contact as are household contacts. Moreover, we believed that football teammates of our case-patients were potentially at greater risk for invasive GAS than typical household contacts because of their greater risk for skin trauma. Any resultant skin disruption could provide a portal of entry for a more virulent GAS strain from an infected teammate, potentially leading to invasive GAS disease even among otherwise healthy persons (*10*).

Because of the severity of the patients’ illnesses and the theoretically increased risk for invasive GAS, we screened and provided antimicrobial prophylaxis to the varsity team during the initial investigation without awaiting final culture results. However, the 8% GAS positivity rate was consistent with published estimates of the overall background colonization rates among schoolchildren (11%–25%) in nonoutbreak settings (*11*–*13*), and lower than the secondary carrier rate among household contacts of persons with streptococcal illness (*14*). Only 1 varsity player carried the same GAS strain as his septicemic teammate. One of the unrelated strains isolated from an asymptomatic player was resistant to erythromycin. This finding emphasizes the possibility that in some situations macrolide antimicrobial agents may not be the most effective for prophylaxis. Given the absence of any additional cases of invasive GAS or increased GAS carriage rates among the varsity team, we screened the junior varsity team but treated only players with positive throat cultures.

Both patients tested positive on rapid streptococcal antigen throat swab tests. Only patient 1 had culture-confirmed *emm*-82 invasive GAS; no positive culture was obtained from patient 2. When patient 2 was assessed for a fluctuant leg mass, he had already received antimicrobial agents for streptococcal pharyngitis. No throat culture was obtained at initial diagnosis, and subsequent leg fluid culture was sterile. Although not approved for testing specimens other than throat swabs, rapid antigen testing (Thermo BioStar Acceava Strep A Test, Thermo Electron Corporation, Louisville CO) of the leg fluid was positive for streptococcal antigen, suggesting that GAS was the etiologic agent. The inability of microbiologic techniques to yield a GAS isolate from patient 2 prevented definitive linkage of the 2 invasive cases. However, the epidemiologic link and temporal proximity of infections suggest that they probably were infected by the same GAS strain. Furthermore, the high sensitivity and specificity of rapid antigen detection in diagnosing GAS pyoderma in children have been demonstrated (*15*). Of note, *emm*-82 accounted for only 3.1% of sterile site isolates in the United States from 1995 to July 2001 (*16*). Thus, even relatively uncommon GAS strains can cause serious infections.

How many cases, if any, might have been prevented by our efforts cannot be determined. However, we received no additional reports of invasive GAS that were epidemiologically linked to this football team in the 10 months after the investigation.

More information is needed regarding the appropriate preventive measures for GAS outbreaks among contacts of invasive GAS patients in athletic settings. Healthcare professionals who care for athletes should be reminded of the potential for outbreaks of infectious diseases. Public health authorities should share their experiences so additional information can be gathered on which to establish consensus guidelines for prevention and control of future invasive GAS clusters or outbreaks occurring among contact sport participants.
